# Bacterial Diversity of Arctic Soils with Long-Standing Pollution by Petroleum Products and Heavy Metals

**DOI:** 10.3390/microorganisms14010055

**Published:** 2025-12-26

**Authors:** Ekaterina M. Semenova, Tamara L. Babich, Diyana S. Sokolova, Vladimir A. Myazin, Maria V. Korneykova, Tamara N. Nazina

**Affiliations:** 1 Winogradsky Institute of Microbiology, Research Center of Biotechnology, Russian Academy of Sciences, 119071 Moscow, Russia; semenova_inmi@mail.ru (E.M.S.); microb101@yandex.ru (T.L.B.); sokolovadiyana@gmail.com (D.S.S.); 2 Institute of North Industrial Ecology Problems–Subdivision of the Federal Research Centre “Kola Science Centre of Russian Academy of Science”, 184209 Apatity, Russia; myazinv@mail.ru; 3 Agrarian and Technological Institute, People’s Friendship University of Russia (RUDN University), 117198 Moscow, Russia

**Keywords:** Arctic soils, hydrocarbons, heavy metals, bacterial diversity, high-throughput sequencing, 16S rRNA gene, bacterial isolation, hydrocarbon-oxidizing bacteria

## Abstract

Long-standing and chronic soil pollution in the Polar Regions is the most persistent. Simultaneous contamination with petroleum products and heavy metals puts additional load on the soil microbial community. The purpose of this work was to determine the composition of prokaryotes in the soils of Mount Kaskama with long-standing contamination with petroleum products and heavy metals (Murmansk region, Russia) and outside this zone and the potential ability of bacteria to participate in the self-purification of these soils. Using high-throughput sequencing of 16S rRNA gene V3–V4 fragments, an increase in the proportion of bacteria of the phyla *Pseudomonadota*, *Verrucomicrobiota*, *Cyanobacteriota*, and *Bacillota* was shown with an increase in soil contamination. Bacteria of the genera *Bacillus*, *Caballeronia*, *Cytobacillus*, *Paenibacillus*, *Paraburkholderia*, *Pseudomonas*, and *Rhodanobacter* were isolated from soil samples. Bacteria of the genus *Paenibacillus* capable of hydrocarbon oxidation and iron reduction were isolated from the subsurface contaminated layers. Under aerobic conditions, Fe(II) oxidation by bacteria of the genus *Pseudomonas* and biodegradation of hydrocarbons by isolated bacteria are possible. The isolated strains grew at low temperatures, used diesel fuel components, and were resistant to Cu(II), Ni(II), and Pb(II). The data obtained indicates the adaptation of bacterial communities to environmental conditions and the ability to participate in the process of soil self-healing.

## 1. Introduction

The Arctic is often called the “World’s treasure trove” due to the vast reserves of mineral resources hidden here. Oil, gas, rare earth metals, diamonds, etc., are recovered in Russian Arctic region. Intensive industrial activity leads to emergency situations and the accumulation of environmental damage [1]. Hydrocarbons, including aliphatic and aromatic hydrocarbons, resins and asphaltenes, have toxic effects on humans, animals, plants, and microorganisms. Hydrocarbon pollution leads to a decrease in biodiversity and ecosystem functioning [2]. The elimination of pollution from petroleum products and heavy metals is an important task for the preservation of this vulnerable region. Modern technologies of soil purification include physical and chemical methods, which are not always applicable in the conditions of the North due to harsh climate and imperfect logistics [3]. Microorganisms play key roles in degradation of hydrocarbon pollution in soil and water ecosystems. Despite extreme climatic conditions, viable and metabolically active microorganisms are found in glaciers, ice, and Arctic soils. Moreover, the microbial diversity in these soils is comparable to that in other regions [4,5,6,7,8,9,10]. A high abundance of oligotrophic bacteria is typical for the soils of the northern regions [7].

Bioremediation is considered an eco-friendly, economical, and sometimes the only possible method to restore polluted soils in the Polar regions [11]. It is the most effective method, leading to the complete mineralization of hydrocarbons [12]. The possibilities of bioremediation, including biostimulation (fertilization, moistening, aeration) and bioaugmentation (introduction of additional oil-degrading bacteria), are described in detail in reviews [13,14]. Adverse environmental conditions, a short growing season, and low fetilty soils determine the persistence of anthropogenic pollution and extremely low rates of soil self-purification in the Polar regions. Decreasing temperature leads to an increase in the viscosity of spilled oil and delayed evaporation of low-molecular-weight toxic compounds, which slows down the microbial degradation of hydrocarbons [15]. Although microbial activity has been proven even at subzero temperatures, the rate of hydrocarbon biodegradation under such conditions is extremely low [16,17,18]. The ability of soil microbiota to resist oil pollution depends on the type of pollutant and the duration of exposure [19]. Over time, the pollutant penetrates into the pores of soil aggregates, its polymerization occurs, and it reacts with soil humus, complicating the biodegradation process [20,21]. In Antarctic soils, hydrocarbons persisted and were detected more than 40 years after contamination [4].

When soils are contaminated simultaneously with petroleum products and heavy metals, microorganisms are subjected to double stress and are forced to adapt to several additional aggressive factors. The toxicity of hydrocarbons is caused by their ability to accumulate and disrupt the cell membranes of microorganisms [22,23]. The simultaneous effect of petroleum products and heavy metals on the microbial community has been poorly studied. The bioavailability of hydrocarbons for microorganisms is not uniform and can be arranged as follows: linear alkanes > branched alkanes > small aromatics > cyclic alkanes [13]. While hydrocarbons can be completely degraded by microorganisms to carbon dioxide and water, heavy metals remain in the soil. The mechanisms of interaction between microorganisms and metals can be different, associated with cellular metabolism and specific enzymes (intracellular separation and extracellular precipitation) or simple physicochemical interactions (adsorption on cell wall components) [24,25]. Microorganisms are capable of participating in the accumulation and sorption of heavy metals, affecting their solubility, toxicity, and mobility [26,27]. The combined use of bacteria and plants promotes the conversion of heavy metals into safer forms and their removal from soils *in situ*, which is important for soil restoration in hard-to-reach places [28]. Iron-reducing microorganisms possess enzymes that reduce heavy metals and metalloids such as U(VI), Tc(VIII), Cr(VI), and Co(III), using acetate, lactate, pyruvate, and aromatic hydrocarbons as substrates and donors of electrons [29,30,31]. The ability to grow on hydrocarbons with the reduction in heavy metals has been shown for bacteria of the genera *Geobacter* and *Rhizobium* [32,33].

The presence of anthropogenic pollutants in the soil leads to changes in the composition of the microbial community, an increase in the proportion of groups tolerant to it, and the disappearance of sensitive ones. Soil pollution with heavy metals leads to the inhibition of microorganism growth and hydrocarbon biodegradation [34,35,36].

The choice of remediation technology used depends on the characteristics of the soils, the pollutant, and environmental conditions [37,38]. The rate of polycyclic aromatic hydrocarbon (PAH) degradation depends on the type of hydrocarbons, the target oil-degrading strain, or consortium of strains [39]. The selection of fertilizers for Northern soils, initially poor in nitrogen and phosphorus, has shown that oligotrophic biostimulation is more effective than eutrophic approaches [40].

Under laboratory conditions, it was shown that the most effective method for cleaning soil from PAHs was a combination of biostimulation and bioaugmentation [41]. In addition to fertilizers, an enrichment culture of oil-oxidizing bacteria isolated from the same soil was introduced, which allowed for the removal of 87% of the pollutant in 80 days, while self-purification achieved only 42%. Heavy metals had less effect on the microbial community than PAHs in soils with aged PAHs and metals contamination [42].

In this work, the bacteria of the hydrocarbon-contaminated soil of Mount Kaskama, located in the Subarctic region (Russia), were studied. Our working hypothesis was the assumption that, despite difficult climatic conditions and heavy hydrocarbon pollution, soil microorganisms adapt to these conditions and participate in the degradation of pollutants, contributing to the self-cleaning ability of soils.

The goal of the present work was to determine the composition of prokaryotes in Russian Subarctic soil with long-standing pollution by petroleum products and heavy metals and outside this zone, and the potential ability of prokaryotes to participate in the self-purification of the soil.

## 2. Materials and Methods

### 2.1. Site Description and Soil Sampling

Soil samples were collected on 7 October 2022, and 2 August 2023, on the southeastern slope of Mount Kaskama (N 69°16′44″; E 29°28′33″; Pechengsky District, Murmansk Region, Russia). Mount Kaskama is located in the subarctic climate zone, and the sampling site, considering altitudinal zonation, can be classified as a mountain tundra zone [43]. The height of the sampling point was about 300 m; it was located 50 m downhill from the top. Soil on the Kaskama mountain slope is represented by podzolized podburs (according to the Russian classification [44]). Under the International Soil Classification, the soil is classified as Entic Podzols [45]. As a result of territory pollution, the plant cover has been destroyed over a large area: on the summit and on sections of varying sizes on the western and eastern slopes.

**Figure 1:**
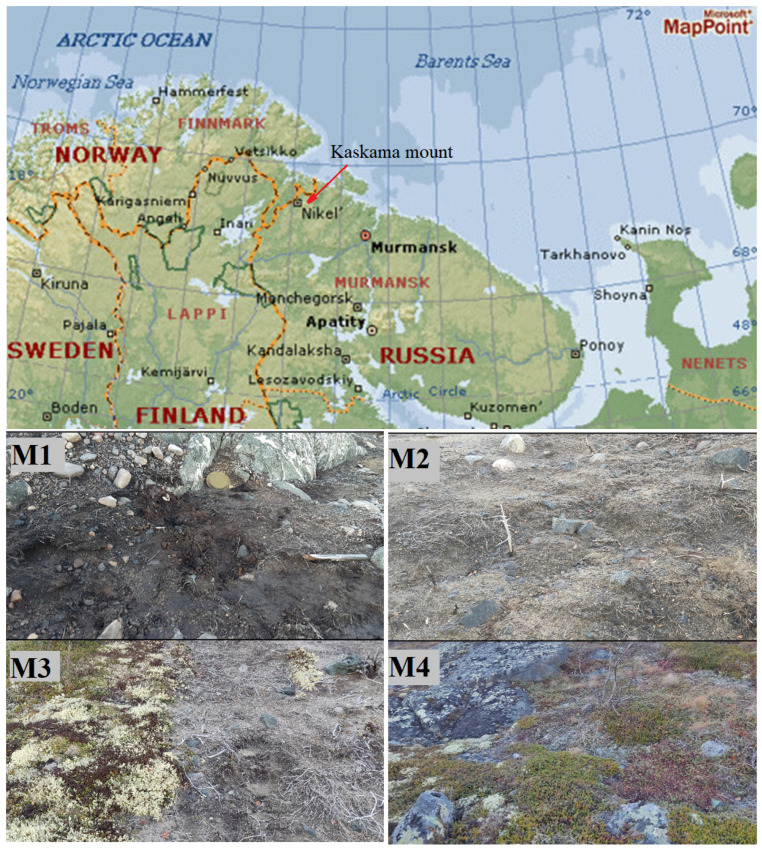
Location map of the Mount Kaskama (**upper** figure) and general view of the M1–M4 sampling sites in 2023 (**lower** figure). Samples were obtained from a depth of 0–15/20 cm at the hydrocarbon and heavy metal contaminated M1–M3 sites and at a visually clean control M4 site.

As a result of anthropogenic impact, a zone of long-standing contamination (over 20 years) with petroleum products (presumably fuels and lubricants), scrap metal, and fragments of equipment had formed. Four sites were selected—from the center of the visible fuel pollution towards the periphery, arranged in a horizontal line approximately 10 m apart from each other (Figure 1). Sites No. 1 and No. 2 were located directly within the contaminated area, site No. 3 was on the border of the contaminated and conditionally clean zone, and site No. 4 was on the conditionally clean zone (with no visible fuel contamination and with preserved vegetation cover). In 2022, samples were collected from depths of 0–10 cm and 10–15/20 cm from the surface (at the boundary with the underlying rocks) aseptically into sterile containers using the X-shaped sampling method. In 2023, samples were collected from a depth of 0–20 cm. The collected samples were stored 2 days at +4 °C until analysis.

### 2.2. Analytical Methods

The gas phase composition (CO_2_, N_2_, N_2_O, and H_2_) in cultures was determined using a “Kristall 5000.2” chromatograph (“Khromatek”, Moscow, Russia).

The actual acidity of the soil and soil moisture were determined as described previously [43]. All analyses were performed immediately upon arrival at the laboratory.

The total petroleum hydrocarbons (TPHs) were extracted from a soil sample with tetrachlorocarbon; the extract was purified on a column with aluminum oxide and then examined by using the AN-2 analyzer (LLC Neftekhimavtomatika, Saint Petersburg, Russia) [43]. The utilization of oil was determined by the change in the composition of the aliphatic fraction of oil compared to the control (in %) using a Crystal 5000.1 gas–liquid chromatograph (Khromatek, Yoshkar-Ola, Russia), with a ZB-FFAP 15 m capillary column and a flame ionization detector, as described earlier [46].

The total content of heavy metals was determined by atomic absorption spectrometry using a spectrometer AAS “Kvant-2M” (Kortek, Moscow, Russia) after microwave digestion of the soil sample in a mixture of concentrated hydrochloric acid, nitric acid, and hydrofluoric acid (MVI 80–2008).

### 2.3. Quantification of Culturable Bacterial Groups and Isolation of Pure Cultures

The number of microorganisms was determined by the tenfold serial dilution method on selective media. The average weight of the soil sample (10 g) was mixed with 90 mL of sterile tap water and shaken for 30 min at 110 rpm. After precipitation of large particles for 1–2 min, the resulting aqueous suspension was used for inoculation of selective media. Aerobic organotrophic bacteria (AOB) were enumerated in a liquid R2A medium (per liter distilled water): 0.5 g peptone; 0.5 g yeast extract; 0.5 g glucose; 0.5 g casein hydrolysate; 0.5 g starch; 0.3 g Na pyruvate; 0.3 g K_2_HPO_4_; 0.024 g MgSO_4_·7H_2_O; 1.0 g NaCl; pH 6.0 ± 0.2 [47]. Oligotrophic bacteria (OL) were enumerated on a medium (per liter distilled water): 0.8 g Na_2_HPO_4_; 0.5 g KH_2_PO_4_; 0.5 g NH_4_Cl; 0.2 g MgSO_4_; 0.1 g CaCl_2_·2H_2_O; 1.0 g NaCl; 0.05 g yeast extract, pH 6.0 ± 0.2. Hydrocarbon-oxidizing bacteria (HOB) were enumerated on a medium (per liter distilled water): 0.75 g KH_2_PO_4_; 1.5 g K_2_HPO_4_; 1.0 g NH_4_Cl; 1.0 g NaCl; 0.1 g KCl; 0.2 g MgSO_4_·7H_2_O; 0.02 g CaCl_2_·2H_2_O, pH 6.0 ± 0.2. After sterilization, 0.1% (*v*/*v*) sterile diesel fuel was added to the medium as a carbon source. To determine the number of iron-oxidizing bacteria (FeOx), a medium [48] was used (per liter distilled water): 0.5 g K_2_HPO_4_; 0.5 g (NH_4_)_2_SO_4_; 0.5 g NaNO_3_; 0.5 g MgSO_4_·7H_2_O; 5.9 g FeSO_4_·7H_2_O; 10.0 g citric acid; 2.0 g sucrose; 1.0 g tryptone; pH 6.6–6.8.

Anaerobic microorganisms were cultivated in Hungate’s tubes. Argon was used as the gas phase. Fermentative bacteria were enumerated on a medium (per liter distilled water): 10.0 g glucose; 4.0 g peptone; 2.0 g Na_2_SO_4_; 1.0 g MgSO_4_; 0.5 g FeSO_4_(NH_4_)_2_SO_4_·6H_2_O; pH 6.0 ± 0.2. For iron-reducing bacteria (FeRed), a medium [49] of the following composition was used (per liter distilled water): 1.5 g NH_4_Cl; 1.0 g NaCl; 0.75 g KH_2_PO_4_; 1.5 g K_2_HPO_4_; 0.1 g CaCl_2_·2H_2_O; 0.6 g NaH_2_PO_4_·H_2_O; 0.1 g MgCl_2_·6H_2_O; 0.1 g KCl; 0.005 g MnCl_2_·4H_2_O; 0.001 g Na_2_MoO_4_; 2.5 g NaHCO_3_ 16.2 g ferric citrate; 2.0 g Na acetate; 5.0 g yeast extract; pH 7.0 ± 0.2. A trace elements solution (1 mL·L^−1^) [50] was added to each medium after sterilization. All experiments were performed in triplicate. Cultivation was carried out for 14 days at 15 °C.

The growth of AOB and OL was determined by changes in the medium turbidity. The growth of iron-oxidizing bacteria was determined by the color change in the medium from light green to rusty brown. The growth of fermentative bacteria was assessed by the increase in H_2_ and CO_2_ in the gas phase. The growth of iron-reducing bacteria was detected by a decrease in Fe^3+^ and an increase in Fe^2+^ content, determined by the method of complexometric titration with sulfosalicylic acid. Abundance, cells morphology and motility of bacteria in cultures were monitored using light microscopy.

Pure cultures of AOB were isolated on R2A agar medium. The temperature range for bacterial growth was determined on R2A medium, and the salinity range was determined on R2A medium at NaCl concentrations from 0% to 10% with a 1% interval. The growth of iron-reducing bacteria on hydrocarbons was determined on the medium [49] without yeast extract and acetate, supplemented with diesel fuel (2 g·L^−1^). Bacterial growth on crude oil and other substrates was determined on the mineral medium for HOB; sugars and protein substrates (sucrose, peptone) were added at a concentration of 5 g·L^−1^, salts of organic acids and alcohols (acetate, ethanol, glycerol)—2.5 g·L^−1^, crude oil and diesel fuel—2.0 g·L^−1^. Growth in the presence of heavy metals was determined in liquid R2A medium; heavy metals were added as NiCl_2_·2H_2_O, CuSO_4_·2H_2_O, and Pb(NO_3_)_2_ to final concentrations of Ni^2+^—40 μg·L^−1^, Cu^2+^—75 μg·L^−1^, and Pb^2+^—100 μg·L^−1^. The effect of metals on bacterial growth was evaluated (in %) by the ratio of turbidity in cultures grown on R2A medium without heavy metals and with metals.

### 2.4. DNA Isolation and the 16S rRNA Gene V3–V4 Fragments Sequencing

DNA from pure cultures was isolated using the Fast DNA Spin Kit (MPBio, Solon, OH, USA), followed by amplification of the 16S rRNA gene with universal primers 8–27f and 1492r [51]. Sequencing was performed on a 3730 DNA Analyzer using the BigDye^®^ Terminator v3.1 Cycle Sequencing Kits (Applied Biosystems, Waltham, MA, USA). Assembly and analysis of the obtained sequences were performed using the Bioedit package (https://bioedit.software.informer.com/, accessed on 17 May 2022). The nearly complete 16S rRNA gene sequences of the isolates were used for phylogenetic identification by BLAST analysis against the GenBank/EMBL/DDBJ databases, using sequences from validly published type strains as references. BLAST algorithm rRNA/ITS databases/16S ribosomal RNA sequences of bacteria and archaea was used for identification (NCBI server, www.ncbi.nlm.nih.gov/blast/, accessed on 17 September 2025).

For DNA isolation from soil samples, the DNeasy PowerSoil Pro DNA isolation kit (QIAGEN, Hilden, Germany) was used according to the manufacturer’s instructions and an average weight of the soil sample (10 g). The PCR amplification of 16S rRNA gene fragments comprising the V3–V4 variable regions was carried out using the universal prokaryotic primers 341F_Fr (5′-CCT AYG GGD BGC WSC AG-3′) and 806R_Fr (5′-GGA CTA CNV GGG THT CTA AT-3′) [52]. The PCR fragments were barcoded using the Nextera XT Index Kit v.2 (Illumina, San Diego, CA, USA) and purified using Agencourt AMPure beads (Beckman Coulter, Brea, CA, USA). The concentrations of PCR products were calculated using the Qubit dsDNA HS Assay Kit (Invitrogen, Carlsbad, CA, USA). All PCR fragments were then mixed and sequenced on FastaSeq300 (GeneMind, Shenzhen, China) platform (2 × 300 nt from both ends).

### 2.5. Data Analysis

All sequence reads were processed using the SILVA pipeline (SILVAngs 1.4) [53]. Each read was aligned using the SILVA Incremental Aligner [54] against the SILVA SSU rRNA SEED and quality controlled [53]. Reads shorter than 50 nucleotides were excluded from further analysis. After these quality control, identical reads were identified and clustered (OTUs) on a per sample basis. The quality of the obtained V3–V4 region sequences was analyzed using UPARSE algorithm [55] of the USEARCH v12 software (https://github.com/rcedgar/usearch12, accessed 14 June 2024) was used for OTUs clustering with ≥97% similarity [56]. The OTUs were taxonomically identified using SILVA v.138 rRNA sequence database and the VSEARCH v. 2.14.1 algorithm (SILVA release 138.1) [57]. The classification was performed by BLASTn (2.17.0+; http://blast.ncbi.nlm.nih.gov/Blast.cgi) [58] with standard settings using the non-redundant version of the SILVA SSU Ref dataset as classification reference (release 138.2; http://www.arb-silva.de). Chao1, Shannon, and Simpson alpha diversity indices were obtained in SILVAngs to characterize the richness and evenness of the bacterial communities. A heatmap of community members at the genus level was constructed using the ClustVis online resource (http://biit.cs.ut.ee/clustvis/, accessed 6 November 2024) [59]. Canonical correspondence analysis (CCA) was performed in the PAST 4.17 program [60]. Venn diagrams were constructed using the online tool InteractiVenn (http://www.interactivenn.net/, accessed 6 November 2025) [61].

### 2.6. Nucleotide Sequence Accession Number

The 16S rRNA gene V3–V4 fragment sequences of M1–M4 microbial communities have been deposited in the NCBI Sequence Read Archive (SRA) and are available via the BioProject PRJNA1346952. Nucleotide sequences of the 16S rRNA gene of pure cultures were deposited into GenBank under accession nos: PX457869, PX457873, PX464108, PX457728, PX462097, PX462100, PX462107, PX462110, PX457785, PX463341, PX463725–PX463727, PX457871, PX457722, PX457868, PX460839, PX457726, and PX463338.

## 3. Results and Discussion

### 3.1. Physicochemical Characteristics of the Soil Samples and Culturable Bacteria

The physicochemical parameters of the soils sampled in different climatic seasons (October 2022 and August 2023) are shown in Table 1. The measured humidity and temperature values corresponded to the climatic norms of the Murmansk region. The temperature in October was 11–12 °C lower than in August. Soil moisture increased significantly in autumn (and exceeded 80%); this indicator was highest in the area with maximum hydrocarbon pollution (M22-1–M22-3 sites). The pH values of the aqueous soil extracts ranged from 4.33 to 6.39 in October and 5.75–5.80 in August, which may be related to the precipitation regime. Petroleum products were found in all the samples studied, even in a visually clean area (M22-4). In 2022, the total petroleum hydrocarbons (TPH) concentration in the upper layer of Site 4 (M22-4-(0-10)) slightly exceeded the approximate permissible concentration (APC) according Russian Regulations, but was significantly lower than at sites M22-1–M22-3.

The content of petroleum products in the lower soil layer was higher than on the surface, with the exception of site M22-4. A similar distribution and seepage of hydrocarbons (HC) deep into the soil has previously been noted by researchers [6]. The binding of HC to the soil matrix decreases their bioavailability and makes soil restoration more difficult. Changes in the TPH concentration in 2022 and 2023 can be caused by the process of soil self-purification, a certain mobility of the pollutant in mountainous conditions, and heterogeneity of pollution.

The content of heavy metals in almost all M1–M4 sites was higher of approximate permissible concentration. The Cd content was more than 3 times higher, and for Cu and Ni it was more than 2 times higher than the corresponding value of the APC. The visually clean site M4 was also significantly polluted with Cu and Ni, and the Cd content in was also higher than APC value in 0–10 cm soil layer. But in the 10–20 cm soil layer of site M4, the content of heavy metals was below the APC.

The number of culturable bacteria of the main physiological groups (Figure S1) was determined in all selected soil samples. It is known that a change in season leads to changes in the composition and abundance of microorganisms in the soil, with temperature and humidity being the main factors [7,8]. On average, the microbial community was more numerous in soil samples taken in August, when the air temperature was 16–17 °C, than in soils taken in October, at 4–5 °C. In the 2023 samples, the abundance of FeOx and fermentative bacteria was highest in the most polluted M1 sample and decreased as it moved away from the contaminated zone. The data obtained are consistent with those presented earlier [10]. A change in the sampling depth of 0–10 and 10–20 cm did not lead to a significant change in the number and redistribution of the physiological groups of the studied microorganisms.

### 3.2. Prokaryotic Diversity

The composition of prokaryotes was determined in soil samples, taken in 2023 year, using high-throughput of 16S rRNA gene V3–V4 fragments sequencing. Among the studied samples, the alpha diversity was slightly higher in the uncontaminated M4 site, as evidenced by a higher Shannon index compared to other soil samples (Table S1). It is likely that a number of microorganisms are sensitive to contamination by petroleum products and heavy metals. The values of the Shannon index for all samples M1–M4 were comparable and even exceeded the values for other, including Arctic, soils [5,7,42,62]. It seems that the soil community has adapted to pollutants. The presence of numerous minor representatives suggests a significant diversity. Rare taxa (<1% in library of 16S rRNA gene sequences of the soil community) are able to survive under certain conditions even in polluted sites and may play a role in regulating microbial interactions in response to environmental changes [63].

**Figure 2:**
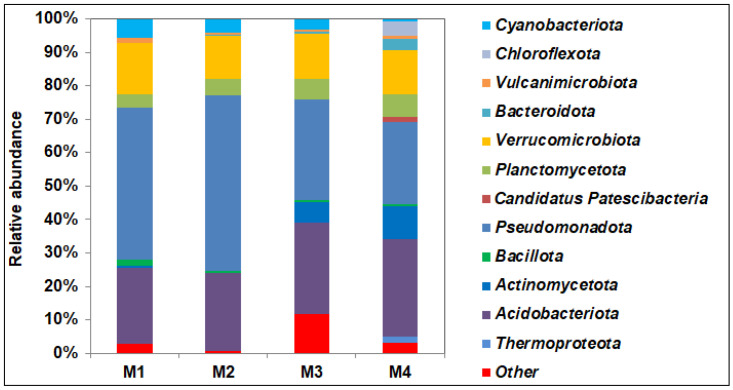
Relative abundance of prokaryotic phyla detected by 16S rRNA gene V3–V4 fragments sequencing in soil samples collected in 2023 at the hydrocarbon and heavy metal contaminated M1-M3 sites and at a control M4 site.

Bacteria dominated in all soil samples analyzed; the proportion of Archaea did not exceed 2% (of the total number of sequences in the library) and was highest in the M4 sample (Figure S2). The taxonomic classification of prokaryotes at the level of orders and families in soil samples is shown in Figure S3a,b. 16S rRNA gene sequencing confirmed community shift in the contaminated soil. An increase in the content of petroleum products in the soil led to a significant increase in the proportion bacteria of the phyla *Pseudomonadota* (from 23.6 to 45.4–52.5%), *Verrucomicrobiota* (from 13.2 to 15.4%), *Cyanobacteriota* (from 0.7 to 5.7%), and *Bacillota* (from 0.4 to 1.72) and a decrease in *Acidobacteriota*, *Actinomycetota*, *Planctomycetota*, *Thermoproteota*, *Bacteroidota*, and *Candidatus* Patescibacteria (Figure 2).

Despite the fact that the phylum *Actinomycetota* contains active hydrocarbon degraders, its share in the M1 sample is significantly lower than that of the phylum *Pseudomonadota*, whose representatives apparently gained an advantage here due to rapid growth and broad metabolic abilities. This distribution was previously detected in soils with long-standing creosote contamination [64]. The predominance of proteobacteria over actinobacteria in the community was noted also in soils in cold regions [65,66]. The predominance of *Pseudomonas* in soil microbial communities, in response to pollution by hydrocarbons and heavy metals has been shown in laboratory conditions [63].

In accordance with the slightly acidic conditions in the soils, the bacteria of the *Acidobacteriota* phylum adapted to these conditions prevailed in the microbial community. It is one of the most widespread soil bacterial phyla found worldwide, from tropical agricultural to Arctic soils and sphagnum peat bogs [67,68,69]. *Acidobacteriota* have been found in microbial communities of bottom sediments of reservoirs contaminated with uranium (U) [70]. For chronically polluted soils around the oldest oil wells in Poland, as well as in the communities we studied, there was a decrease in the proportion of *Acidobacteriota* as pollution increased [71]. The authors found the greatest diversity in communities of samples taken directly near the well, where *Mycobacteriaceae*, *Methylococcaceae*, *Bradyrhizobiaceae*, *Rhizobiaceae*, *Rhodobacteraceae*, *Acetobacteraceae*, *Hyphomicrobiaceae*, and *Sphingomonadaceae* bacteria dominated.

**Figure 3:**
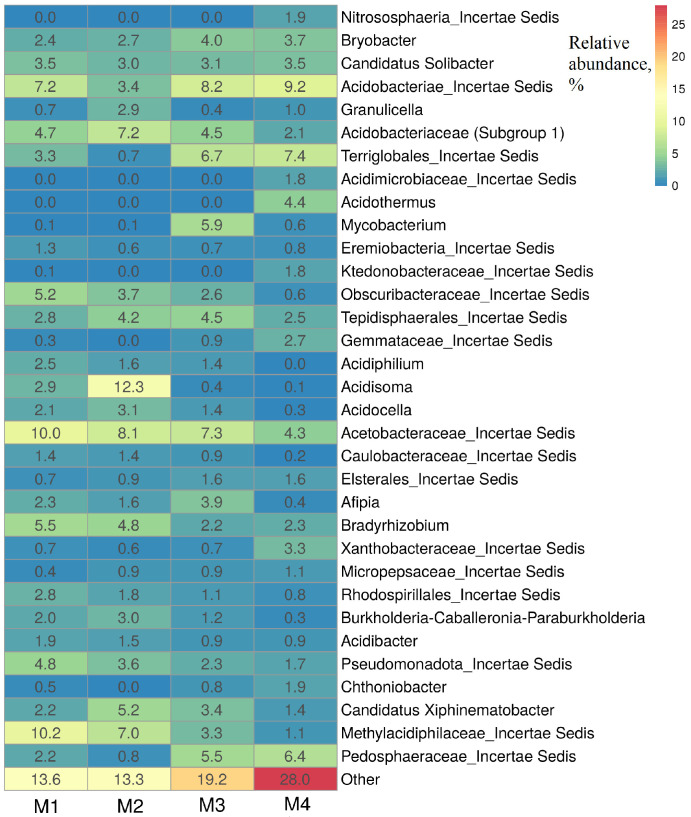
Heatmap based on 16S rRNA gene amplicon sequencing showing the relative abundance (%) of taxonomic groups of prokaryotes in contaminated M1–M3 sites and at a visually clean control M4 site. The numbers in the diagram indicate % of the total number of sequences in the library from each soil sample studied. The taxa with a relative abundance of ≥1.1% in at least one library are listed.

In the M1–M4 soil samples were detected unidentified bacteria of the families *Acetobacteraceae*, *Acidobacteraceae*, *Methylacidiphilaceae*, as well as bacteria of the genera *Acidisoma*, *Bryobacter*, *Acidothermus*, *Acidocella*, *Acidiphilum* and others (Figure 3).

These bacteria include acidophilic and acidotolerant heterotrophic bacteria capable of growing at low temperatures, such as *Acidisoma* bacteria isolated from acidic tundra soil [72]. Bacteria of the genus *Bryobacter*, identified in all M1–M4 samples, are chemoorganotrophs, which were previously isolated from acidic sphagnum peat bogs [67].

In the studied soil samples the increase in the *Pseudomonadota* was not due to the rapidly growing *Gammaproteobacteria*, which includes the most famous oil-degrading bacteria of the genera *Pseudomonas*, *Marinobacter*, and *Alkanivorax* but due to *Alphaproteobacteria* (genera *Acidiphilum*, *Acidisoma*, *Acidocella*, and *Bradyrhizobium*). These bacteria are adapted to inhabit acidic soils, mines, and swamps [72]. Bacteria of the genus *Acidiphilum* are resistant to the presence of heavy metals [73]. Strains of *Acidocella aromatica* are able to grow on phenol, reduce Fe^3+^, and resistant to heavy metals [74]. Bacteria of the genus *Bradyrhizobium* are capable of using hydrocarbons and resistant to heavy metals and pesticides. They have been found in microbial communities of oil-contaminated soils and contain genes determining the aliphatic and aromatic hydrocarbons oxidation [75,76,77,78,79]. The *Burkholderia fungorum* FM-2 strain, which oxidizes phenanthrene in a wide range of pH values and resistant to heavy metals, was isolated from the oil-contaminated soil of an oil field in China [36]. Microbial communities of the studied soils also contained unidentified bacteria. The search for conditions for the isolation and research of such microorganisms will make it possible to better understand the functioning of the entire soil community of Polar soils [80].

The number of 16S rRNA gene sequence libraries was insufficient for analysis of the core microbiome to identify microbial groups shared among contaminated and control soils and representing organisms highly adapted to the environmental and nutritional conditions. However, Venn diagrams show the number and proportion of shared and unique phyla between the libraries of bacterial 16S rRNA genes from studied soil samples. About 48.5% of nucleotide sequences in libraries were affiliated with bacteria of 16 phyla and were detected both in contaminated M1–M3 soil samples and in a control M4 soil (Figure S3).

**Figure 4:**
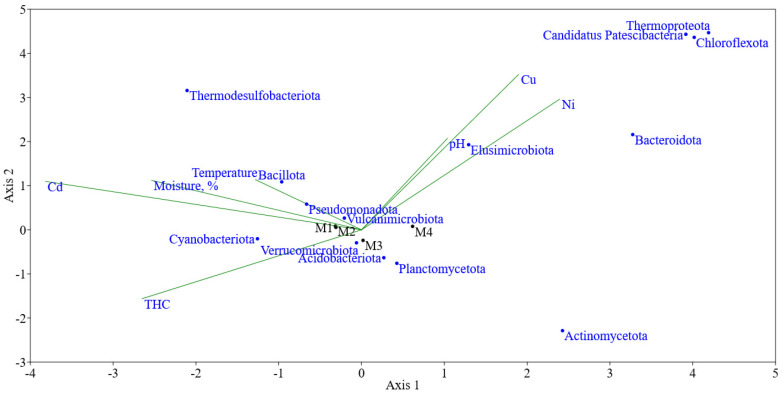
Canonical correspondence analysis (CCA) of 16S rRNA gene-based microbial community composition in highly contaminated M1–M3 soil samples and in a clean control M4 soil as well as environmental parameters, including the total petroleum hydrocarbons (TPH) and Cu, Ni, and Cd contamination, soil moisture, pH and temperature. Green lines indicate the direction of microbial composition associated with environmental parameters.

Canonical correspondence analysis showed that a positive correlation can be traced for the content of hydrocarbons in the soil, probably as a substrate used (Figure 4). If we consider the effects of heavy metals, the correlation was positive for Cu and Ni, and negative for Cd, which is associated with its higher toxicity. Previously, for soils chronically subjected to hydrocarbon and polymetallic contamination, it was shown that PAHs rather than heavy metals have a greater impact on the community [42].

### 3.3. Culturable Hydrocarbon-Oxidizing Bacteria Isolated from Contaminated Soil

A total of 20 strains of aerobic heterotrophic bacteria were isolated from soil samples taken in 2022–2023 years (Table S2). Three isolated strains were Gram-positive bacteria of the genera *Bacillus*, *Cytobacillus*, and *Paenibacillus*. They belonged to the *Bacillota* phylum and were found among the minor components of the M1–M4 soil communities. The other 17 strains were Gram-negative bacteria of the *Pseudomonadota* phylum and were affiliated with the classes *Betaproteobacteria* (genera *Caballeronia* and *Paraburkholderia*) and *Gammaproteobacteria* (genera *Pseudomonas* and *Rhodanobacter*). Eleven of twenty isolated strains belonged to the genus *Pseudomonas*, which can be explained by their high growth rate under specified laboratory conditions.

For further work, 10 strains effectively degrading diesel fuel were selected and their adaptability to the environment was determined, including the temperature range for growth, the use of hydrocarbons and resistance to heavy metals. All the studied strains of the genera *Pseudomonas*, *Caballeronia*, *Rhodanobacter*, and *Paraburkholderia* were psychrotolerant, able to grow at a temperature of 5 °C and at an upper temperature limit of 30–37 °C. The temperature growth range for *R. ginsengisoli* M23-91 was at 5–30 °C. The strains grew at low optimal NaCl content for growth (0–2%, *w*/*v*) (Table 2). The substrates used included carbohydrates, alcohols, and volatile fatty acids. The ability to oxidize divalent iron has been shown for *Pseudomonas* strains. The strains were able to grow on diesel fuel and crude oil. Most strains used medium-chain n-alkanes; *P. yamanorum* M22-22H and *P. fluorescens* M23-K6fo strains used both medium-chain and long-chain n-alkanes from crude oil (Figure S5).

Bacteria of the genus *Pseudomonas* have a flexible metabolism, use a wide range of substrates (including hydrocarbons), and are highly resistant to heavy metals. These bacteria have been found in soils of different geographical regions and have found application in many biotechnologies [81]. Bacteria of the genera *Rhodococcus*, *Pseudomonas*, and *Bacillus* have been repeatedly found in oil-contaminated polar soils of the Arctic and Antarctica [82].

Bacteria of the genus *Caballeronia*, phylogenetically close to *Burkholderia*, have been isolated from various types of soil and rhizosphere, they grow in a wide range of pH values (from 4.0 to 10.0) and are capable of degrading aromatic compounds and xenobiotics [83,84]. A strain of *Paraburkholderia* (*Pb.*) *fungorum* JT-M8 is described, capable of self-regulation of Cd ion concentration in the cytoplasm and on the cell surface under conditions of simultaneous contamination with PAHs and heavy metals (Cd) with phosphorus deficiency [85]. Bacteria of the genus *Rhodanobacter* are tolerant to low pH values and to contamination with heavy metals, including nickel, copper, and cadmium [86,87,88]. They are found in microbial communities that degrade crude oil [89]. In the microbial community of oil-contaminated soils of the subarctic zone, *Rhodanobacter ginsengisoli* was one of the dominant oil degraders [90]. Spore-forming bacteria of the genera *Bacillus* and *Cytobacillus* are common inhabitants of soils, including the soil of the Polar region [10,13,91].

Previously, the ability of the indigenous soil microfungi and bacteria for hydrocarbon degradation was demonstrated in a field experiment on the western slope of Mount Kaskama [43], where aeration and fertilization of the oil-contaminated soil led to a 47% reduction in total hydrocarbon content.

**Figure 5:**
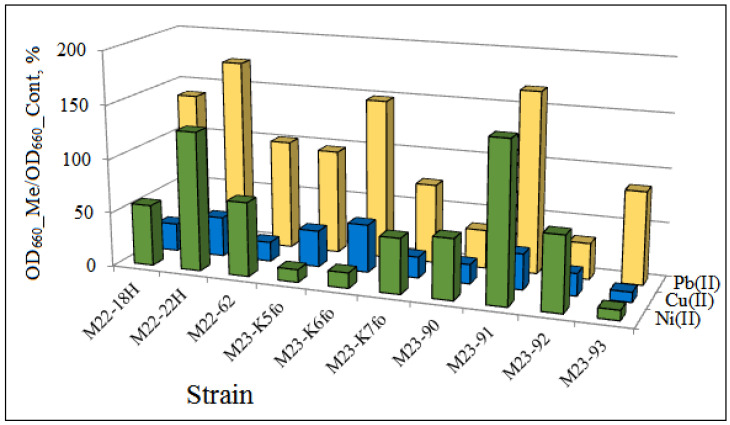
The resistance of soil isolates to Ni, Pb and Cu contamination calculated as the ratio of strain growth in a medium with metals (OD_660__Me) to the growth in a control medium without metals (OD_660__Cont) in %. Strains: *P. hamedanensis* M22-18H, *P. yamanorum* M22-22H, *P. synxantha* M22-62, *P. frederiksbergensis* M23-K5fo, *P. fluorescens* M23-K6fo, *P. synxantha* M23-K7fo, *C. sordidicola* M23-90, *R. ginsengisoli* M23-91, *C. udeis* M23-92, and *Pb. domus* M23-93.

Laboratory analysis revealed the content of heavy metals (nickel, copper, and cadmium) in M1–M3 soil samples exceeded the approximate permissible concentration. The toxicity of heavy metals occurs in low concentrations of about 1.0–10 mg·L^−1^ while some metals ions such as Chromium (Cr), Lead (Pb), Zinc (Zn), Copper (Cu), Nickel (Ni), Cadmium (Cd), etc., are very toxic in lower concentration of 0.001–0.1 mg·L^−1^ [91]. The maximum tolerance concentration to heavy metals for the isolated bacterial strains was not determined in this study. The presence of 40 μg·Ni^2+^ L^−1^ in a medium stimulated the growth of two strains, *P. yamanorum* M22-22H and *R. ginsengisoli* M23-91 (Figure 5). The Cu^2+^ concentration used (75 μg·L^−1^) suppressed the growth of all 10 strains studied. Five strains, M22-18H, M22-22H, M23-K6fo, and M23-91, were insensitive to Pb^2+^ at a concentration of 100 μg·L^−1^. The resistance of the isolates to heavy metals decreased in the range Pb(II) > Ni(II) > Cu(II), which is comparable with data of other researchers [91]. However, the maximum tolerance concentration to heavy metals of bacteria of the genera *Pseudomonas*, *Cupriavidus*, *Bacillus*, and *Acinetobacter* [91,92] was essentially higher of metal concentrations used in our study. The studied microorganisms are adapted to inhabit soils slightly polluted with heavy metals. These results indicate the adaptation of the isolated strains to the conditions of their habitat and their possible participation in the process of soil self-purification.

### 3.4. Culturable Fe(III)-Reducing Bacteria

Both aerobic and anaerobic processes are possible in the 0–20 cm soil layer. An analysis of the number and composition of communities revealed the presence of both aerobic hydrocarbon- and iron-oxidizing, as well as anaerobic or facultatively anaerobic bacteria capable of fermentation and reduction of metals. The processes of metal oxidation and reduction can occur in parallel in microzones with different oxygen contents [93]. Bacteria involved in both the oxidation process and the reduction process in the iron cycle have been described [94]. Bacteria capable of Fe^3+^ reduction can also reduce other metals (Mn^4+^, for example) and vice versa [24,95], therefore ferric citrate was chosen as a model compound. The poly-contamination of soils with petroleum products and heavy metals prompted us to check whether HC oxidation is possible in these microbial communities, coupled with the Fe^3+^ reduction. A similar process has been shown for bacteria of the genera *Geobacter* and *Rhizobium* [24,33].

Stable enrichment cultures were obtained from M2 and M3 soil samples, which carried out the process of Fe^3+^ reduction to Fe^2+^, and oxidation of the alkane fraction (Figure S6). Enrichment culture M2 used medium-chain alkanes to a greater extent, while culture M3 used medium- and long-chain alkanes. The low hydrocarbon biodegradation is probably due to the fact that the rate of anaerobic degradation of hydrocarbons is significantly lower than the aerobic HCs oxidation. Since many iron-reducing bacteria are able to grow aerobically, the resulting enrichment cultures were inoculated on plates with a solid nutrient medium, from which it was possible to isolate the FeRed2 and FeRed3 strains. The isolated strains were able to reduce Fe^3+^, as well as grow aerobically on diesel fuel and crude oil (Figure S7), and were identified as *Paenibacillus pseudetheri* and *Paenibacillus nitricinens*, respectively (Table S2) [96,97].

Bacteria of the genus *Paenibacillus* are able to grow on aromatic hydrocarbons and have previously been isolated from Arctic soils contaminated with petroleum products [10,98]. These bacteria are known for their ability to reduce and sorb heavy metals, form siderophores and form floccules, which allow them to be used for water purification from heavy metals [99,100,101]. *Paenibacillus* spp. demonstrated high resistance to heavy metals (Cd) [102]. Probably, the bacteria of the genus *Paenibacillus* isolated from the studied soils are able to participate in the process of soil self-purification both in the aerobic and anaerobic zones, using anaerobic respiration for the degradation of hydrocarbons.

Isolation of psychrophilic bacteria, which effectively oxidize hydrocarbons and their use for bioaugmentation of northern soils, will reduce the time of soil remediation from hydrocarbon pollution. The obtained results showed that the presence of two types of pollutants, such as hydrocarbons and heavy metals, is capable of initiating both aerobic and anaerobic processes of hydrocarbon biodegradation, accompanied by metal reduction. Thus, the self-cleaning zone for petroleum products can be expanded, although the rate of aerobic oxidation is undoubtedly higher than that of the anaerobic process. The study of the mechanisms of bacterial adaptation to environmental conditions will allow a deeper understanding of the ability and patterns of participation of the microbial communities in the process of soil self-purification.

## 4. Conclusions

Remediation of long-standing complex soil pollutants in Polar Regions is an important and difficult task. This work shows that a multifunctional autochthonous prokaryotic community capable of biodegradation of hydrocarbons, oxidation and reduction in metals inhabit the soils of Polar Regions with long-standing pollution by petroleum products and heavy metals. The total content of hydrocarbons and heavy metals influenced the composition of bacterial communities. An increase in the content of petroleum products in the soil led to a significant increase in the bacteria of the phyla *Pseudomonadota*, *Verrucomicrobiota*, *Cyanobacteriota*, and *Bacillota* and to a decrease in *Acidobacteriota*, *Chloroflexota*, *Actinomycetota*, *Planctomycetota*, *Thermoproteota*, *Bacteroidota*, and *Candidatus* Patescibacteria. The increase in the *Pseudomonadota* was due to *Alphaproteobacteria* (genera *Acidiphilum*, *Acidisoma*, *Acidocella*, and *Bradyrhizobium*) apparently adapted to the conditions of poor northern soils. Culturable bacteria capable of degrading hydrocarbons and oxidizing metals were isolated from the upper aerobic zone, while bacteria capable of reducing Fe^3+^ for growth on hydrocarbons were present in the anaerobic subsurface zone. Isolated pure cultures of bacteria of the genera *Bacillus*, *Caballeronia*, *Cytobacillus*, *Paenibacillus*, *Paraburkholderia*, *Pseudomonas*, and *Rhodanobacter* degraded petroleum n-alkanes. Strains of the genus *Pseudomonas* oxidized Fe^2+^, and *Paenibacillus* spp. were capable of reducing Fe^3+^ to Fe^2+^, which allows them to participate in the process of soil self-purification. The isolated strains are resistant to the used concentrations of heavy metals; their toxicity decreased in the range Cu^2+^ > Ni^2+^ > Pb^2+^. Long-term microbiological monitoring is necessary to develop effective strategies to eliminate long-term soil contamination. Heavy metal contamination can stimulate the degradation of petroleum products in the anaerobic zone through metal reduction, thereby reducing metal mobility. This contributes to both a decrease in hydrocarbon content and the immobilization of heavy metals. The data obtained indicate the potential of autochthonous bacteria for remediation of the subarctic contaminated soils.

## Figures and Tables

**Table 1:** Physicochemical characteristics and the total petroleum hydrocarbons (TPH) and metal content in soil samples.

Year of Sampling, Site	Depth of Sampling, cm	Temperature, °C	Soil Humidity, %	pH of the Aqueous Soil Extract	TPH Content, mg/kg	Cu^2+^, mg/kg	Ni^2+^, mg/kg	Cd^2+^, mg/kg
2022, October								
M22-1-(0–10)	0–10	4.8	>80	4.49	52,729	40.11	23.85	1.423
M22-1-(10–20)	10–20	5.1	>80	4.37	64,530	27.91	22.84	1.446
M22-2-(0–10)	0–10	5.0	39	4.65	54,182	55.50	35.56	1.924
M22-2-(10–20)	10–20	4.8	46	4.28	115,079	16.55	16.07	1.244
M22-3-(0–10)	0–10	4.4	27	4.33	29,305	14.76	19.58	1.494
M22-3-(10–15)	10–15	4.7	20	4.46	34,008	19.50	24.13	0.522
M22-4-(0–10)	0–10	4.9	23	5.90	890	75.85	40.94	1.226
M22-4-(10–15)	10–15	5.0	32	6.39	183	21.20	15.61	0.191
2023, August								
M1	0–20	17.6	58	5.80	52,500	34.01	23.34	1.44
M2	0–20	16.0	40	5.75	14,900	36.02	25.82	1.584
M3	0–20	16.1	37	5.75	37,700	17.03	21.86	1.008
M4	0–15	16.3	35	5.79	530	48.73	28.28	0.701
APC *					700	33.00	20.00	0.500
Standard error		±0.1	±3.1	±0.03	±5.0	±0.01	±0.01	±0.001

* APC, approximate permissible concentration.

**Table 2:** Physiological characteristics of strains isolated from polluted soils.

No.	Strain	Temperature Optimum, °C	NaCl Range (Optimum), %	Substrate *	Fe^2+^ Oxidation to Fe^3+^
1	*Pseudomonas hamedanensis* M22-18H	15–20	0–3.5 (1)	Ac, Eth, Gly, Suc, Pept	+ **
2	*Pseudomonas yamanorum* M22-22H	15–25	0–8 (1–2)	Ac, Eth, Gly, Pept	+
3	*Pseudomonas synxantha* M22-62	20–30	0–5 (2)	Ac, Eth, Gly, Suc, Pept	+
4	*Pseudomonas frederiksbergensis* M23-K5fo	20–25	0–7 (0–1)	Ac, Eth, Gly, Suc, Pept	+
5	*Pseudomonas fluorescens* M23-K6fo	20–25	0–8 (0–1)	Ac, Gly, Pept	+
6	*Pseudomonas synxantha* M23-K7fo	20–25	0–5 (2)	Ac, Eth, Gly, Suc, Pept	+
7	*Caballeronia sordidicola* M23-90	15–30	0–6 (0)	Gly, Pept	−
8	*Rhodanobacter ginsengisoli* M23-91	20	0–1 (0–0.5)	Eth, Gly, Suc, Pep	−
9	*Caballeronia udeis* M23-92	20	0–5 (0)	Ac, Gly, Pep	−
10	*Paraburkholderia domus* M23-93	25–28	0–5 (0)	Ac, Gly, Pep	−

Designations: * Acetate—Ac; ethanol—Eth; glycerol—Gly; sucrose—Suc; peptone—Pept. ** +, positive result; −, negative result.

## Data Availability

The 16S rRNA gene sequences of isolated strains presented in the study are openly available in GenBank under accession Nos: PX457869, PX457873, PX464108, PX457728, PX462097, PX462100, PX462107, PX462110, PX457785, PX463341, PX463725–PX463727, PX457871, PX457722, PX457868, PX460839, PX457726, and PX463338. The raw data generated from 16S rRNA gene profiling of M1–M4 microbial communities presented in the study are openly available in NCBI Sequence Read Archive (SRA) via the BioProject PRJNA1346952.

## Supplementary Materials

The following supporting information can be downloaded at: https://www.mdpi.com/article/10.3390/microorganisms14010055/s1, Figure S1: The number of culturable microorganisms in soil samples in October 2022 (a) and August 2023 (b); Figure S2: Taxonomic classification of prokaryotes at the level of the Bacteria and Archaea domains in soil samples based on the high-throughput sequencing of 16S rRNA genes in soil samples collected at the hydrocarbon and heavy metal contaminated M1–M3 soil samples and at a visually clean control M4 soil; Figure S3: Taxonomic classification of prokaryotes at the order (a) and family (b) level, based on the high-throughput sequencing of 16S rRNA genes, in soil samples collected at the hydrocarbon and heavy metal contaminated M1–M3 soil samples and at a control M4 soil; Figure S4: Venn diagrams showing the number and proportion of shared and unique phyla between the libraries of bacterial 16S rRNA genes from contaminated M1–M3 soil samples and a control M4 soil; Figure S5: Residual content of n-alkanes in crude oil (in % relative to the content of n-alkanes in the sterile control) degraded by *Pseudomonas hamedanensis* M22-18H (a), *Pseudomonas yamanorum* M22-22H (b), *Pseudomonas synxantha* M22-62 (c), *Caballeronia sordidicola* M23-90 (d), *Caballeronia udeis* M23-92 (e), *Paraburkholderia domus* M23-93 (f), *Pseudomonas frederiksbergensis* M23-K5fo (g), and *Pseudomonas fluorescens* M23-K6fo (h). The bacteria were incubated for 14 days at 15 °C; Figure S6: Residual content of n-alkanes in diesel fuel (in % relative to the content of n-alkanes in the sterile control) degraded by Fe^3+^-reducing enrichment from M2 (a) and M3 (b) soil samples on the medium with Fe^3+^ citrate and diesel fuel. The enrichments were incubated for 60 days at 15 °C; Figure S7: Residual content of n-alkanes in crude oil (in % relative to the content of n-alkanes in the sterile control) degraded by strain *Paenibacillus pseudetheri* FeRed2 (a) and *Paenibacillus nitricinens* FeRed3 (b). The cultures were incubated aerobically with sterile crude oil for 21 days at 23 °C; Table S1: Diversity indices in V3–V4 libraries of prokaryotic 16S rRNA gene fragments from the studied soil samples; Table S2: Taxonomic affiliation of the isolated strains based on the analysis of 16S rRNA genes.
