# Supplementary material for: Bacterial Diversity of Arctic Soils with Long-Standing Pollution by Petroleum Products and Heavy Metals
Source: Microorganisms. 2025 Dec 26;14(1):55. doi: 10.3390/microorganisms14010055 (PMC12843735; doi:10.3390/microorganisms14010055)
Supplement: Supplementary file 1 [file microorganisms-14-00055-s001.zip › microorganisms-4016807-supplementary.pdf]

microorganisms
MDPI

Supplementary Materials

Bacterial Diversity of Arctic Soils with Long-standing Pollution by Petroleum Products and Heavy Metals

Ekaterina M. Semenova [1], Tamara L. Babich [1], Diyana S. Sokolova [1], Vladimir A. Myazin [2,3], Maria V. Korneykova [2,3,*], and Tamara N. Nazina [1,*]

[1] Winogradsky Institute of Microbiology, Research Center of Biotechnology of the Russian Academy of Sciences, 119071 Moscow, Russia; semenova_inmi@mail.ru (E.M.S.); microb101@yandex.ru (T.L.B.); sokolovadiyana@gmail.com (D.S.S.); nazina@inmi.ru (T.N.N.)
[2] Institute of North Industrial Ecology Problems–Subdivision of the Federal Research Centre "Kola Science Centre of Russian Academy of Science", 184209 Apatity, Russia; myazinv@mail.ru (V.A.M.); korneykova.maria@mail.ru (M.V.K.)
[3] Agrarian and Technological Institute, People's Friendship University of Russia (RUDN University), 117198 Moscow, Russia; myazinv@mail.ru (V.A.M.); korneykova.maria@mail.ru (M.V.K.)
* Correspondence: nazina@inmi.ru; Tel.: +7-499-135-0341 (T.N.N.); korneykova.maria@mail.ru; Tel.: +7-921-288-8830 (M.V.K.)

This file includes:

Figures S1 to S7

Tables S1 and S2

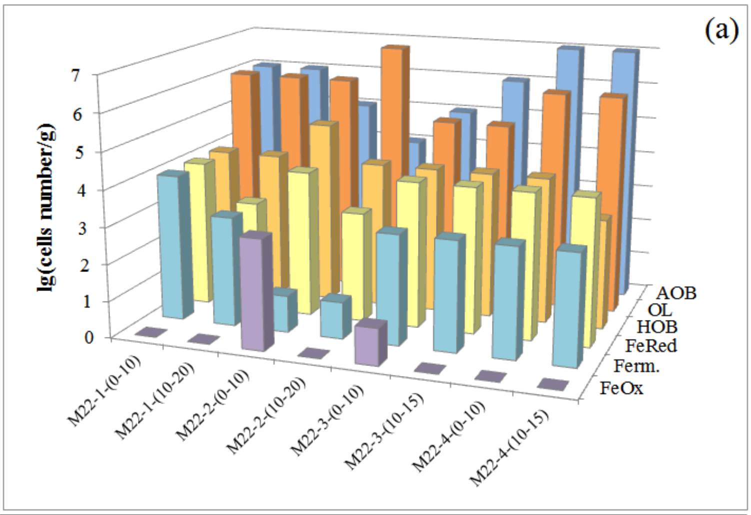

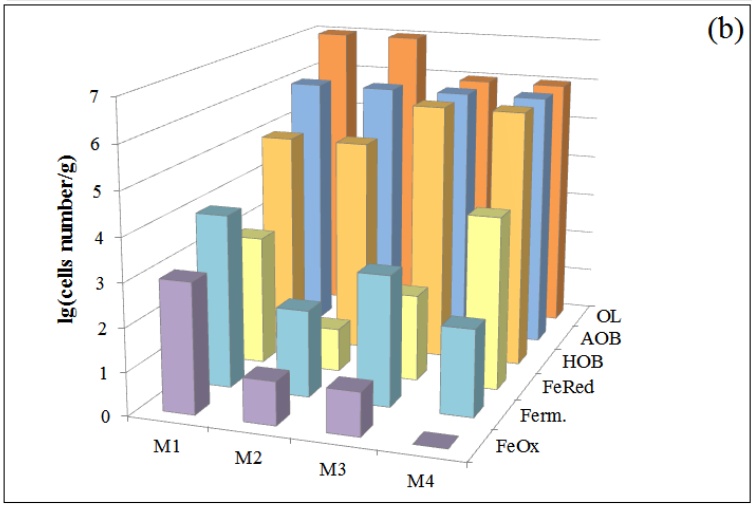

Figure S1. The number of culturable microorganisms in soil samples in October 2022 (a) and August 2023 (b).

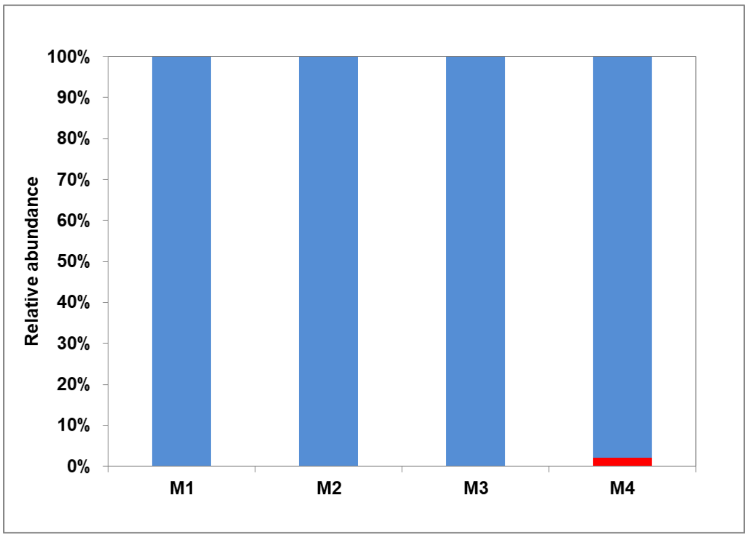

Figure S2. Taxonomic classification of prokaryotes at the level of the *Bacteria* and *Archaea* domains based on the high-throughput sequencing of 16S rRNA genes in soil samples collected at the hydrocarbon and heavy metal contaminated M1–M3 soil samples and at a visually clean control M4 soil.

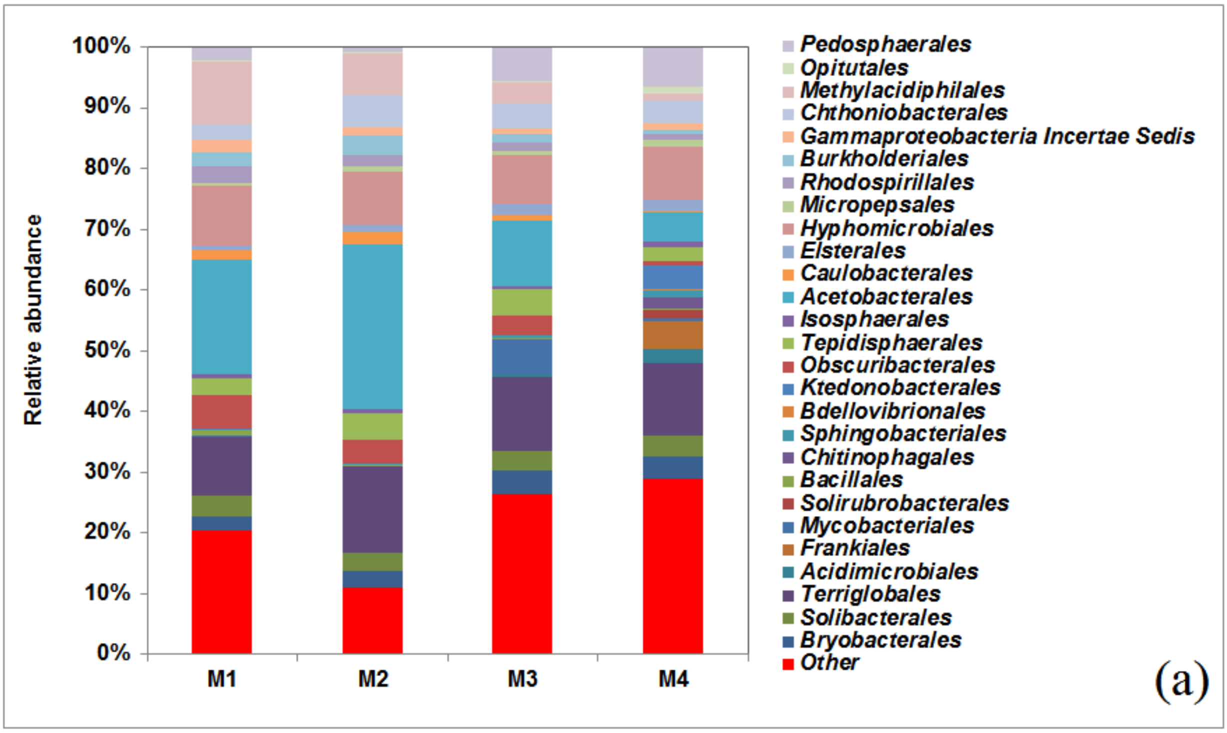

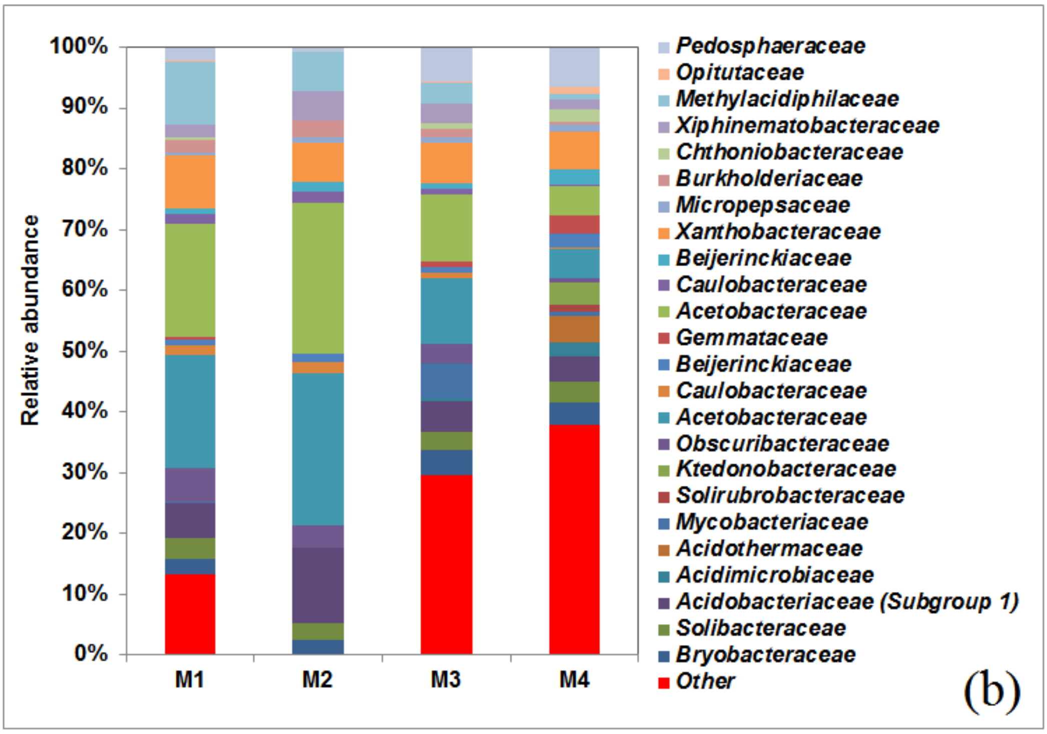

Figure S3. Taxonomic classification of prokaryotes at the order (a) and family (b) level, based on the high-throughput sequencing of 16S rRNA genes, in soil samples collected at the hydrocarbon and heavy metal contaminated M1–M3 soil samples and at a control M4 soil.

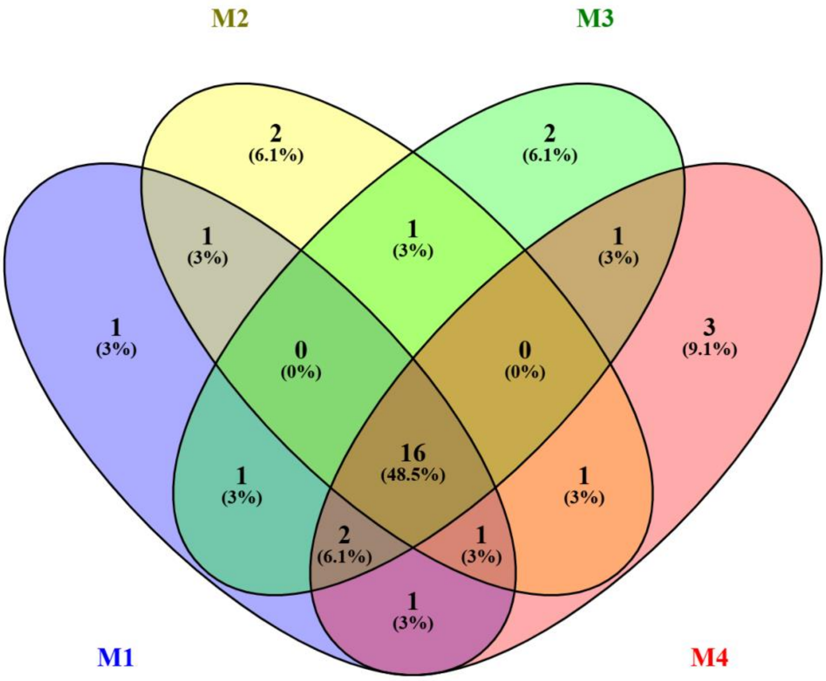

Figure S4. Venn diagrams showing the number and proportion of shared and unique phyla between the libraries of bacterial 16S rRNA genes from contaminated M1–M3 soil samples and a control M4 soil.

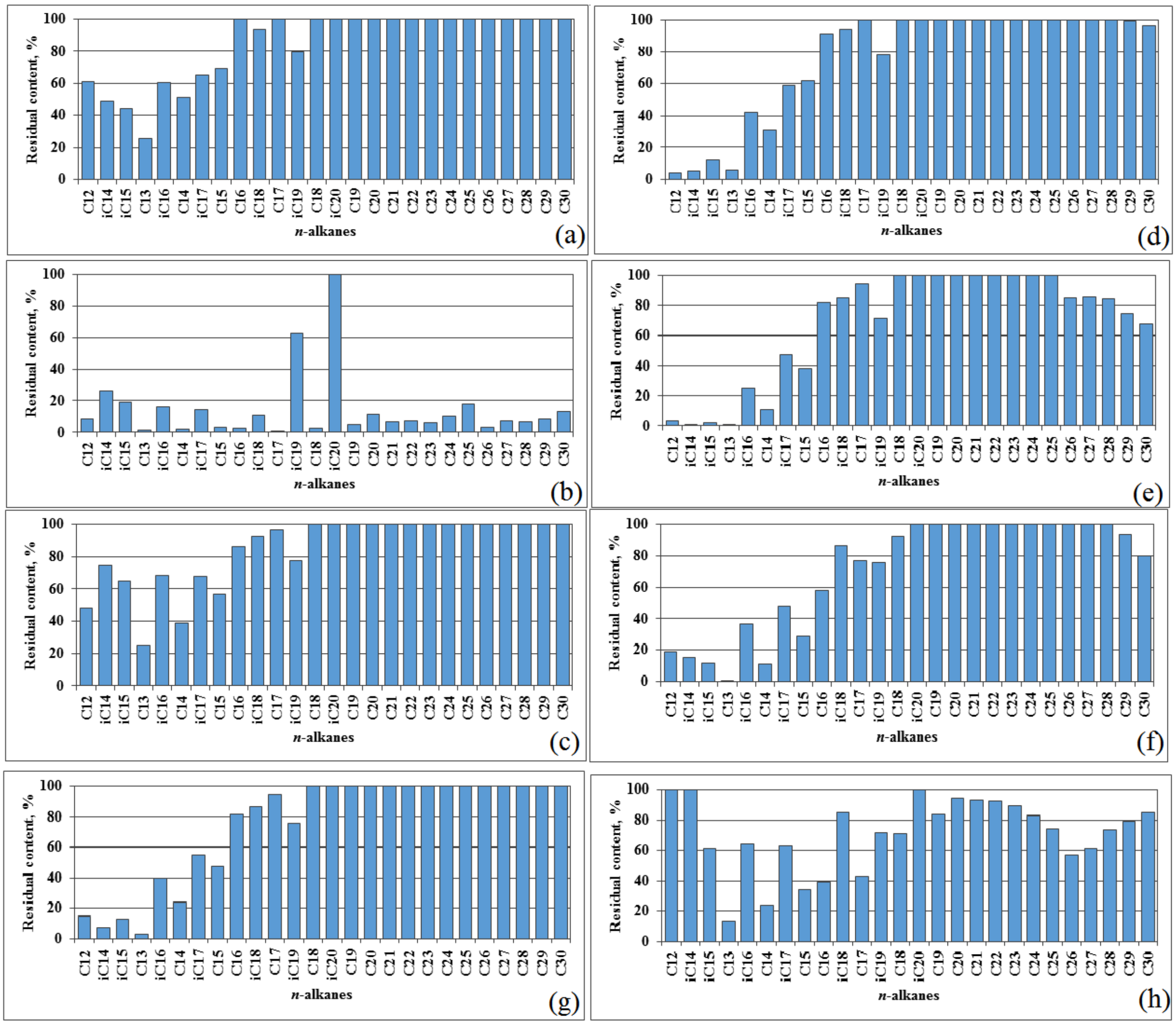

Figure S5. Residual content of *n*-alkanes in crude oil (in % relative to the content of *n*-alkanes in the sterile control) degraded by *Pseudomonas hamedanensis* M22-18H (a), *Pseudomonas yamanorum* M22-22H (b), *Pseudomonas synxantha* M22-62 (c), *Caballeronia sordidicola* M23-90 (d), *Caballeronia udeis* M23-92 (e), *Paraburkholderia domus* M23-93 (f), *Pseudomonas frederiksbergensis* M23-K5fo (g), and *Pseudomonas fluorescens* M23-K6fo (h). The bacteria were incubated for 14 days at 15 °C.

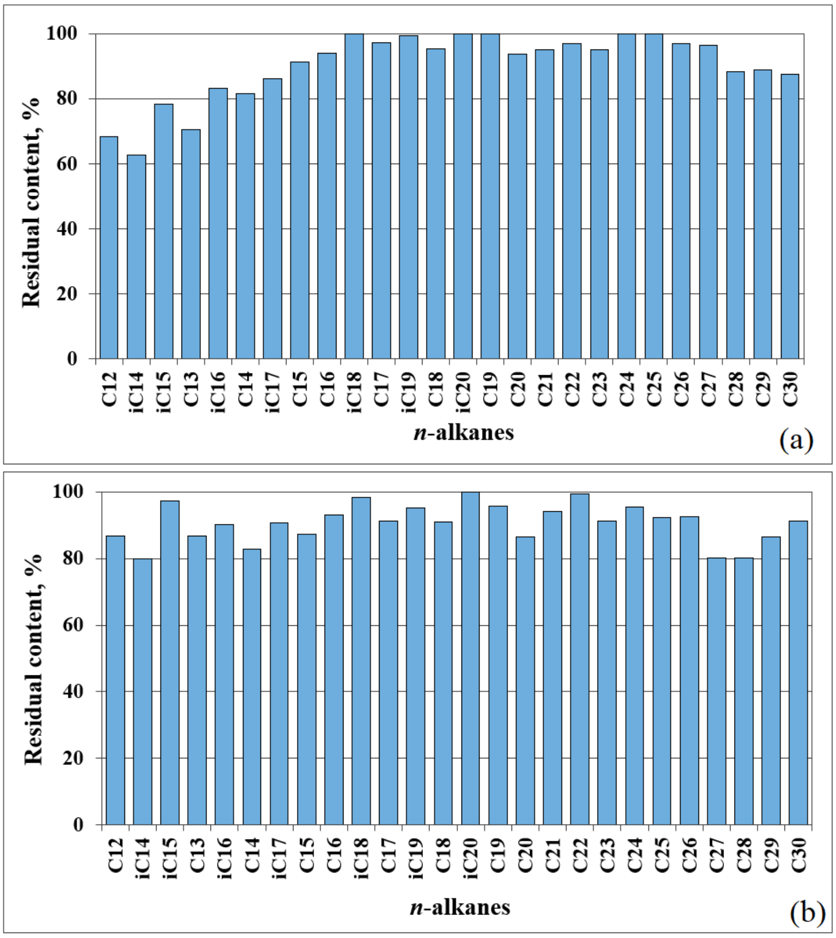

Figure S6. Residual content of *n*-alkanes in diesel fuel (in % relative to the content of *n*-alkanes in the sterile control) degraded by Fe^{3+}-reducing enrichment from M2 (a) and M3 (b) soil samples on the medium with Fe^{3+} citrate and diesel fuel. The enrichments were incubated for 60 days at 15 °C.

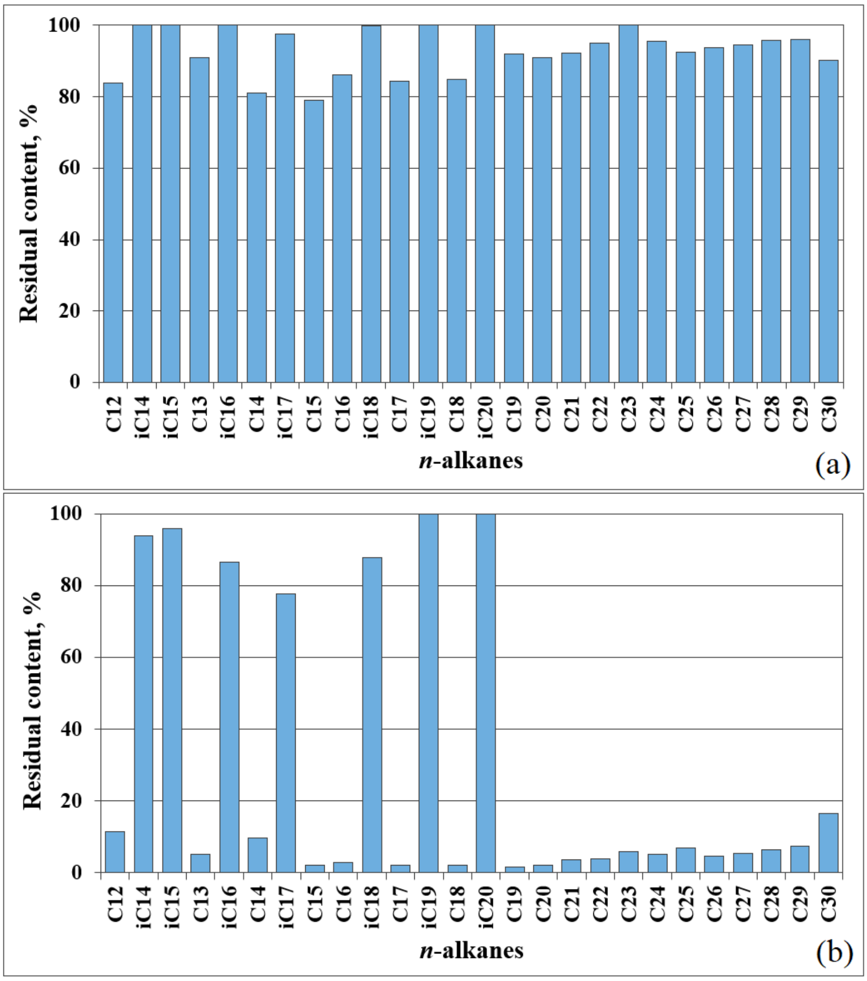

Figure S7. Residual content of *n*-alkanes in crude oil (in % relative to the content of *n*-alkanes in the sterile control) degraded by strain *Paenibacillus pseudetheri* FeRed2 (a) and *Paenibacillus nitricinens* FeRed3 (b). The cultures were incubated aerobically with sterile crude oil for 21 days at 23 °C.

Table S1. Diversity indices in V3–V4 libraries of prokaryotic 16S rRNA gene fragments from the studied soil samples.

Sample	Number of reads	OTUs number	Chao1 index	Good's coverage of library	Shannon index (H10)	Simpson index (1-D)
M1	38295	16586	114693.4	0.63	8.33	0.05
M2	39466	16596	116047.3	0.64	8.36	0.05
M3	45173	14418	95828.06	0.74	7.82	0.05
M4	31653	16727	97939.66	0.56	8.95	0.03

Table S2. Taxonomic affiliation of the isolated strains based on the analysis of 16S rRNA genes.

No.	Strain	The closest species in the GeneBank, strain, 16S rRNA gene accession number	16S rRNA gene similarity, %	The 16S rRNA gene accession number	Sampling site
1	M22–78	*Bacillus mycoides* NBRC 101228; NR_113990.1	100	PX457869	M22-3-(10–15)
2	M23–90	*Caballeronia sordidicola* LMG 22029; MW227315.1	99.3	PX457873	M1-(0–20)
3	M23–92	*Caballeronia udeis* strain Hg2 MN727307.1	99.4	PX464108	M2-(0–20)
4	M22–27H	*Cytobacillus oceanisediminis* H2; OL875278.1	100	PX457728	M22-2-(10–20)
5	FeRed2	*Paenibacillus pseudetheri* JJ-60; OP341378.1	99.6	PX463726	M1-(10–20)
6	FeRed3	*Paenibacillus nitricinens* AC7; CP183948.1	99.8	PX463727	M2-(0–10)
7	M22–79	*Paenibacillus odorifer* DSM 15391; MW228038.1	99.0	PX462097	M22-3-(10–15)
8	M23–93	*Paraburkholderia domus* LMG 31832; NR_178457.1	99.9	PX462100	M4-(0–15)
9	M23–K7fo	*Pseudomonas synxantha* NCTC10696, D84025.1	99.1	PX462107	M3-(0–20)
10	M22–85	*Pseudomonas edaphica* RD25, NR_179227.1	99.9	PX462110	M22-4-(0–10)
11	M22–64	*Pseudomonas fluorescens* JCM 5963, LC462170.1	100	PX457785	M22-1-(0–10)
12	M22–68	*Pseudomonas fluorescens* JCM 5963, LC462170.1	100	PX463341	M22-1-(10–20)
13	M23–K6fo	*Pseudomonas fluorescens* DSM 50090, MT605329.1	100	PX463725	M4-(0–15)
14	M23–K5fo	*Pseudomonas frederiksbergensis* DSM 13022; NR_117177.1	99.9	PX457871	M4-(0–15)
15	M22–18H	*Pseudomonas hamedanensis* SWRI65; CP077091.1	99.6	PX457722	M22-2-(10–20)
16	M22–62	*Pseudomonas synxantha* NCTC10696; D84025.1	98.6	PX457772	M22-1-(0–10)
17	M22–67	*Pseudomonas yamanorum* 8H1; NR_178342.1	100	PX457868	M22-1-(10–20)
18	M22–16H	*Pseudomonas yamanorum* 8H1; NR_178342.1	100	PX460839	M22-1-(10–20)
19	M22–22H	*Pseudomonas yamanorum* 8H1; NR_178342.1	100	PX457726	M22-3-(10–15)
20	M23–91	*Rhodanobacter ginsengisoli* GR17-7; NR_044127.1	98.5	PX463338	M1-(0–20)
